# Impact of Medical Therapy on Atheroma Volume Measured by Different Cardiovascular Imaging Modalities

**DOI:** 10.4061/2010/134564

**Published:** 2010-07-01

**Authors:** Mohamad C. N. Sinno, Mouaz Al-Mallah

**Affiliations:** Heart and Vascular Institute, Henry Ford Hospital, Detroit, MI 48202, USA

## Abstract

Atherosclerosis is a systemic disease that affects most vascular beds. The gold standard of atherosclerosis imaging has been invasive intravascular ultrasound (IVUS). Newer noninvasive imaging modalities like B-mode ultrasound, cardiac computed tomography (CT), positron emission tomography (PET), and magnetic resonance imaging (MRI) have been used to assess these vascular territories with high accuracy and reproducibility. These imaging modalities have lately been used for the assessment of the atherosclerotic plaque and the response of its volume to several medical therapies used in the treatment of patients with cardiovascular disease. To study the impact of these medications on atheroma volume progression or regression, imaging modalities have been used on a serial basis providing a unique opportunity to monitor the effect these antiatherosclerotic strategies exert on plaque burden. As a result, studies incorporating serial IVUS imaging, quantitative coronary angiography (QCA), B-mode ultrasound, electron beam computed tomography (EBCT), and dynamic contrast-enhanced magnetic resonance imaging have all been used to evaluate the impact of therapeutic strategies that modify cholesterol and blood pressure on the progression/regression of atherosclerotic plaque. In this review, we intend to summarize the impact of different therapies aimed at halting the progression or even result in regression of atherosclerotic cardiovascular disease evaluated by different imaging modalities.

## 1. Introduction

Atherosclerosis is a systemic disease that can affect multiple vascular beds and is associated with significant mortality and morbidity. There is an increased interest in the cardiovascular (CV) community in studying the impact of medical therapy on the progression or even the regression of atheroma volume and extent. Change in atheroma volume in response to novel therapies is an attractive “surrogate endpoint” for clinical cardiovascular events as it reflects the pathophysiology of the underlying disease, and offers a more economically feasible approach to test efficacy with fewer patients and resources, and over a shorter follow-up duration [1]. The usual hard and soft clinical endpoints have economic and logistical implications [2] and thus CV researchers have always been eager to identify other surrogate endpoints that would correlate with improvement in clinical outcomes. The enthusiasm for measuring plaque volume is also because increments in the size of atherosclerotic plaque correlate with major adverse cardiovascular events (MACE) [3, 4]. Such observations have fueled efforts at studying medications that target plaque regression or decrease progression early on in patients with atherosclerotic coronary artery disease (CAD). This is based on the premise that a favorable effect of novel therapies on atherosclerotic plaque volume would translate into a favorable clinical effect, and help efficiently triage novel therapies from the laboratory bench to the bedside. This process has been facilitated by the development of new imaging techniques that can assess atherosclerotic plaque. A number of imaging modalities that visualize the arterial wall provide a unique opportunity to characterize the impact of potential anti-atherosclerotic therapies in the *in vivo *setting. Herein, we provide a review of medications that target plaque volume. 

## 2. Therapies That Target Atheroma Volume

### 2.1. The Effects of Antihypertensive Agents (Table 1) 

#### 2.1.1. Calcium Channel Blockers

 The potential effect of calcium channel blockers on atherosclerosis has been studied more than 20 years ago. The “regressive” effects of nicardipine and nifedipine on atherosclerosis in cholesterol-fed mice were observed after 8 weeks of treatment by a reduction in aortic arch plaque area and cholesterol accumulation [5]. Waters et al. [6] in 1992 found that nicardipine had no effect on angiographically detected advanced atherosclerosis but may halt the progression of minimal lesions through its antihypertensive effects. Several clinical trials [7–11] that studied the anti-atherosclerotic effects of calcium channel blockers showed regression of carotid intima-media thickness detected by B-mode ultrasonography. The Prospective Randomized Evaluation of the Vascular Effects of Norvasc Trial (PREVENT) [9] randomized 825 patients with nonobstructive CAD to amlodipine versus placebo. At the end of the follow-up period, the progression and development of new atherosclerotic lesions detected by quantitative coronary angiography were similar in the two groups (−0.084 mm versus −0.095 mm, *P* = .38). In the same trial, a subset of patients (*n* = 377), had regression/stabilization of CIMT detected by high resolution B-mode carotid ultrasonography in the amlodipine group, while progression was uninterrupted in the placebo group (−0.013 mm versus +0.033 mm, *P* = .007). The mechanism of amlodipine-associated slowing of the progression of intima-media thickness may be related to its antihypertensive effect, as well as to its effect on cellular growth and hyperplasia of the arterial wall. Likewise, on the other hand, the Coronary AngioPlasty Amlodipine REStenosis Study (CAPARES) [12] investigated the effect of amlodipine versus placebo on minimal luminal diameter detected by quantitative coronary angiography in patients with stable angina pectoris undergoing percutaneous coronary angioplasty. The trial showed that treatment with amplodpine did not affect minimal luminal diameter assessed by quantitative coronary angiography (−0.30  ±  0.45 mm versus −0.29  ±  0.49 mm; *P* = .84) after a four-month period. However, the study showed that the incidence of repeat percutaenous coronary intervention and MACE were significantly lower in patients treated with amlodipine. Similarly, the IVUS-based trial, Comparison of Amlodipine and Enalapril to Limit Occurrence of Thromobosis (CAMELOT) and Norvasc for Regression of Manifest Atherosclerotic Lesions by Intravascular Sonographic Evaluation (NORMALISE) [13, 14] showed a significant reduction in MACE with amlodipine but not with enalapril or placebo. This finding however, did not project to the same extent in the coronary arteries. The percent atheroma volume measured by IVUS in 274 patients was relatively unchanged in the amlodipine group (+0.5  ±  3.9%; *P* = .31), and increased somewhat in the enalapril group, (+0.8  ±  3.7%; *P* = .08), and significantly in the placebo group (+1.3  ±  4.4%; *P* = .001) (all numbers reflect percent change in atheroma volume from baseline after a 24 month period). There was no statistical difference in percent change in atheroma volume across groups.

#### 2.1.2. Angiotensin Converting Enzyme Inhibitors and Angiotensin II Receptor Blockers

The Prevention of Atherosclerosis with Ramipril (PART-2) Collaborative Research Group [15] examined the anti-atherosclerotic effect of ramipril (5–10 mg/d) or placebo in 617 patients with coronary or other occlusive arterial disease. B-mode ultrasonography revealed no structural difference between groups in changes in common carotid artery-wall thickness or in carotid plaque score at 2 and 4 years with a trend toward a benefit in death from cardiovascular events. The Study to Evaluate Carotid Ultrasound changes with Ramipril and Vitamin E (SECURE) [16], a substudy of the Heart Outcomes Prevention Evaluation (HOPE) trial, used B-mode carotid ultrasonography to monitor atherosclerotic lesions in patients aged 55 years or older with vascular disease or diabetes and at least one other risk factor. Ramipril reduced carotid artery atherosclerosis progression rates, as measured by intimal medial thickness. In a quantitative coronary angiography substudy of 450 randomly selected patients from the Quinapril Ischemic Event Trial (QUIET), quinapril did not differ from placebo in progression of coronary atherosclerosis, new stenosis development, change in minimum lumen diameter index, or change in percent diameter stenosis index [17]. Similar results were obtained from another quantitative coronary angiography study, the Simvastatin/Enalapril Coronary Atherosclerosis Trial (SCAT) [18], where Enalapril failed to show regression in atheroma volume, but showed a significantly lower combined endpoint of death/myocardial infarction/stroke than placebo. 

The anti-atherosclerotic effects of Angiotensin II receptor blockers (ARB) were elucidated in animal models [19]. The MORE study [20] used 2D-ultrasound to assess the changes in common carotid intima-media thickness in hypertensive patients treated with olmesartan. Olmesartan significantly reduced the atheroma volume of larger (>33.7 *μ*L) atherosclerotic plaques compared with atenolol (−11.5  ±  4.4 *μ*L versus +0.6 ± 2.5 *μ*L; *P* = .023). The effect of ARB on atheroma volume in coronary arteries was studied in 64 patients with nonocclusive left main CAD [21]. Serial IVUS studies were performed at baseline and after 7-month follow-up. In the ARB group, vessel volume index significantly decreased during follow-up (9.9 ± 3.1 mm^2^ versus 9.1  ±  2.7 mm^2^; *P* < .01). These clinical trials suggest that ARB could cause regression of atherosclerosis in the vascular beds of humans.

#### 2.1.3. *β*-Adrenergic Receptor Blockers

*β*-Adrenergic receptor blockers reduce recurrent myocardial infarction, sudden cardiac death, and all cause mortality in patients after myocardial infarction [22–25]. To study the effect of Beta-Blocking agents on the progression of atherosclerosis, Sipahi et al. [26] conducted a post-hoc, pooled analysis of individual patient data from 4 intravascular ultrasonography (IVUS) trials: Reversal of Atherosclerosis with Aggressive Lipid Lowering (REVERSAL) [14], Acyl-CoA: Cholesterol Acyltransferase Intravascular Atherosclerosis Treatment Evaluation (ACTIVATE) [27], A Study to Evaluate the Effect of Rosuvastatin On Intravascular Ultrasound (ASTEROID) [28], and CAMELOT/NORMALISE [13, 29]. The latter trial was described above and compared the effects of amlodipine to Enlapril and placebo in reducing atheroma volume. The REVERSAL study evaluated the effects of moderate versus intensive lipid-lowering therapy with statins. ACTIVATE evaluated the effect of the acyl coenzyme A (CoA)—cholesterol acyltransferase inhibitor pactimibe, and ASTEROID evaluated the effect of very-high-intensity lipid-lowering therapy with rosuvastatin on the progression rate of coronary atherosclerosis. This pooled analysis of individual data from 1515 patients enrolled in these 4 trials and followed up for 18 to 24 months revealed that atheroma volume decreased significantly in patients receiving *β*-blockers compared to those who did not (−2.4  ±  0.5 mm^3^/yr versus −0.4  ±  0.8 mm^3^/yr; *P* = .034).

#### 2.1.4. Mineralocorticoid Hormones

 Mineralocorticoid hormones play an important role in endothelial dysfunction, vascular fibrosis, and inflammation in the vasculature, and is involved in the pathogenesis of hypertension [30, 31]. Takai et al. studied the anti-atherosclerotic effects of the mineralocorticoid receptor blocker, eplerenone, in nonhuman primates fed a high cholesterol diet [32]. IVUS analysis of the thoracic aorta revealed that the ratio of intimal volume to total volume was significantly lower in a dose-dependent manner in the eplerenone-treated groups. This positive finding in nonhuman primates has not been validated in human vascular beds.

### 2.2. Therapies That Target Cholesterol (Table 2) 

#### 2.2.1. Statins, Niacin, Ezetimibe, Fibrates, and Colestipol

 The direct relation between serum LDL-cholesterol and HDL-cholesterol versus serial changes in coronary plaque dimensions was elucidated in the study by Von Birgelen et al. [33]. Standard IVUS analysis of 60 left-main coronary arteries obtained 18 months apart revealed a positive linear relationship between LDL-cholesterol and annual changes in plaque size. An LDL cholesterol cut-off value of 75 mg/dl was found at which there was no increase in atheroma cross-sectional area. Furthermore, HDL-cholesterol levels had an inverse relationship with changes in plaque size. This correlation between lipoprotein levels and atheroma volume progression/regression pushed cardiovascular researchers to study the effects of serum lipid modification on angiographic endpoints. 

The cholesterol lowering atherosclerosis study (CLAS) [34] evaluated the effect of lipid lowering on structural angiographic endpoints in 162 patients and correlated these outcomes with functional clinical endpoints. The atheroma volume assessed after 2 years of treatment with niacin/colestipol by global change score (GCS) and quantitative coronary angiography revealed the following by GCS (drug versus placebo): regression (16% versus 4%), no change (45% versus 37%), and progression (39% versus 59%) (*P *= .004) and a significant improvement in percent stenosis (0.3  ±  5.9% versus 2.7  ±  5.8%; *P *= .02) and minimum lumen diameter (−0.01  ±  0.22 mm versus −0.09  ±  0.26 mm; *P *= .04) detected by quantitative coronary angiography. The same Simvastatin/Enalapril Coronary Atherosclerosis Trial (SCAT) [18], mentioned above, evaluated the anti-atherosclerotic effects of statins in 394 normocholesterolemic patients over 4 years. Patients taking simvastatin had less progression in their atherosclerotic lesions, highlighted by a 1.67% change in percent diameter stenosis in the simvastatin group versus 3.83% in the placebo group; *P* = .0003 detected by quantitative coronary angiography and less often required percutaneaous coronary intervention during the study period. The anti-atherosclerotic effect of Simvastatin/niacin in patients with low HDL and normal LDL cholesterol was evaluated in 160 patients randomized to 1 of 4 treatment arms by Brown et al. [35]. Coronary angiography repeated after 3 years of therapy showed regression in percent stenosis in proximal coronary arteries in the simvastatin/niacin group compared to placebo (0.4% decrease versus 3.9% increase, *P* < .001). This structural benefit detected on follow-up angiography translated into a lower MACE rate (3% versus 24%, *P* = .04). 

The REVERSAL trial [14] studied the structural effects of intensive lipid lowering therapy with 80 mg atorvastatin versus moderate lipid lowering with 40 mg pravastatin. The baseline LDL-cholesterol was reduced to 110 mg/dL in the pravastatin group and to 79 mg/dL in the atorvastatin group (*P* < .001). The percentage change in atheroma volume from baseline measured in 654 patients with high LDL (mean 150.2 mg/dL) and angiographic CAD (>20% stenosis) was significantly lower in the atorvastatin group, *P* = .02. Atheroma volume increased in the moderate lipid-lowering arm (pravastatin 40 mg) by a mean of 2.4% (95% CI 0.2%–4.7%, *P* = .001) and remained almost the same in the atorvastatin group after an 18-month follow-up (mean decrease by 0.4%, 95% CI −2.4%–1.5%, *P* = .98). 

Other studies demonstrated that LDL-cholesterol lowering with statins could reverse angiographically detected CAD. In the ESTABLISH study, Okazaki et al. [36] analyzed the impact of 20 mg of atorvastatin on nonculprit lesions in patients with acute coronary syndrome by serial IVUS. Plaque volume was significantly reduced in the atorvastatin group (13.1  ±  12.8% decrease) compared with the control group (8.7  ±  14.9% increase; *P* < .0001). This structural change correlated with a significant decrease (41.7%, *P* < .0001) in LDL-cholesterol level by lipid-lowering therapy for 6 months (*r* = 0.612, *P* < .0001). The Low-density Lipopoprotein Apheresis Coronary Morphology and Reserve Trial (LACMART) [37] conducted in patients with familial hypercholesterolemia evaluated the effects of LDL-cholesterol lowering with apheresis on atheroma volume. At one-year follow-up, the medication+LDL-Apheresis (LDL-A) group showed 28.4% reduction in total cholesterol and 34.3% reduction in LDL cholesterol after one-year follow-up, while the medication alone group showed no changes in cholesterol levels. IVUS evaluation at 1 year showed a decrease in plaque area (baseline: 8.45  ±  4.22 mm^2^ versus 1 yr: 7.76  ±  4.34 mm^2^), and an increase in minimal lumen diameter (baseline: 1.99  ±  0.73 mm versus 1 yr: 2.11  ±  0.81 mm)from study outset in the LDL-A group and a reverse result ((baseline plaque area: 7.19  ±  2.88 mm^2^ versus 1 yr plaque area: 8.08  ±  3.14 mm^2^); (baseline MLD: 2.24  ±  0.89 mm versus 1 yr MLD: 2.16  ±  0.84 mm)) in the medication only group (*P* = .008 and.017 for change in plaque area and change in MLD, resp.). Similarly positive results were recently observed in A Study to Evaluate the Effect of Rosuvastatin on Intravascular Ultrasound-Derived Coronary Atheroma Burden (ASTEROID) [28]. Intensive lipid lowering with 40 mg Rosuvastatin in the 349 patients (69%) who had 24-month follow-up [38] caused a decrease in LDL cholesterol by 53.4% and an increase in HDL cholesterol by 14.6% from baseline. Median reduction of total atheroma volume from baseline was 6.8% (*P* < .001) after 24 months of intensive treatment by rosuvastatin. 

The effect of lipid lowering therapy on plaque composition was highlighted in another study that compared the effect of 20 mg atorvastatin versus usual care among patients with coronary artery disease [39]. At 12-month follow-up plaque volume and plaque echogenicity was assessed by IVUS. Mean absolute plaque volume showed a larger increase in the usual care group compared with the atorvastatin group (9.6  ±  28.1 and 1.2  ±  30.4, resp.; *P* = .191). The hyperechogenicity index, a marker of plaque composition, increased to a larger extent for the atorvastatin group than for the usual care group, with a significant treatment effect for the percent change (atorvastatin 42.2%, usual care 10.1%; *P* = .021). 

The Ezetimibe and Simvastatin in Hypercholesterolemia Enhances Atherosclerosis Regression (ENHANCE) trial [40] evaluating the role of 80 mg of simvastatin with or without 10 mg of Ezetimibe in 720 patients with familial hypercholesterolemia revealed that combination therapy did not result in a significant reduction in CIMT after 24 months of therapy (Simvastatin only: 0.0058  ±  0.0037 mm versus 0.0111  ±  0.0038 mm in the simvastatin-plus-ezetimibe; *P* = .29). Another more recent 3-year trial, Stop Atherosclerosis in Native Diabetics Study (SANDS) [41], compared the effect of standard therapy with lifestyle modification  ±  Simvastatin to attain conventional goals for LDL-C (100 mg/dl), non–HDL-C (130 mg/dL), and SBP (130 mm Hg), to aggressive therapy with lifestyle modification  ±  Simvastatin  ±  Ezetimibe to achieve goals of 70 mg/dL, 100 mg/dL, and 115 mm Hg, respectively. By the end of the 3-year period, the CIMT progressed in the standard therapy group (+0.039 mm) and regressed in the aggressive therapy group (−0.025 mm in ezetimibe plus statin versus −0.012 mm in nonezetimibe group), *P* = .0001. There was no additional benefit of adding Ezetimibe to Simvastatin on CIMT regression in patients who achieved their target LDL-C. Measuring Effects on Intima-Media Thickness: An Evaluation of Rosuvastatin (METEOR) study [42], the largest placebo-controlled statin trial evaluating the effects of Rosuvastatin on CIMT in low risk patients (10-year Framingham risk score <10%), showed a significant regression in CIMT compared to placebo (−0.0014 versus 0.0131 mm/yr; *P* < .001) which failed to reflect on a positive clinical cardiovascular outcome. Another intriguing unpublished 1-year clinical trial, the CASHMERE (Carotid Atorvastatin Study in Hyperlipidemic Postmenopausal Women: a Randomised Evaluation of Atorvastatin versus Placebo), evaluating the effect of 80 mg of atorvastatin compared to placebo in 399 post-menopausal women, found no statistical difference in CIMT results. These inconsistent results correlating CIMT with clinical outcomes were a subject of debate lately questioning the patient population being studied or the technique used in measuring CIMT. 

The ARBITER 6-HALTS trial [43] is a more recent controversial study presented at the American Heart Association late breaking trials sessions in 2009 comparing the effects of extended-release niacin to ezetimibe on CIMT progression after 8 and 14 months of treatment in 208 patients at high risk for atherosclerotic vascular disease with LDL-cholesterol levels (<100 mg/dL) and a moderately reduced HDL level (<42 mg/dL). This study showed Niacin to be superior to ezetimibe in affecting the regression of mean and maximal CIMT both at 8 and 14 months of treatment. Besides, niacin showed a progressive regression in CIMT from 8 to 14 months.

#### 2.2.2. Fibrates

 The effect of Fibrate use on the changes in atheroma volume has been highlighted in a few clinical trials. Fenofibrate use in well-controlled diabetics has been shown in the Diabetes Atherosclerosis Intervention Study (DAIS) [44] to slow the progression of coronary atherosclerosis compared to placebo over a 3-year period, measured by QCA (decrease in minimum lumen diameter (−0.06 ± 0.016 mm versus −0.10 ± 0.016 mm, *P* = .029), without a significant improvement in cardiovascular endpoints. In the Fenofibrate Intervention and Event Lowering in Diabetes (FIELD) study [45], Fenofibrate use for 5 years in patients with type-2 diabetes was not associated with an improvement in mean CIMT throughout the study period, *P* = .987. Another study [46] comparing the effect of Fenofibrate on top of antihypertensive therapy to antihypertensive treatment alone on CIMT demonstrated some improvements after 24 months of therapy. CIMT remained the same in both treatment groups, with a significant improvement in CIMT to carotid artery Diameter ratio (CIMT/D) (12.98 ± 2.62 versus 12.12 ± 2.26%), *P* < .05] in the fenofibrate group. This beneficial effect translated into a lower incidence of stroke in the fenofibrate intervention group (11.30% versus 21.82%; *P* < .05). The St. Mary's Ealing, Northwick Park Diabetes Cardiovascular Disease Prevention (SENDCAP) Study [47], studied the effect of a 3-year treatment with Bezafibrate on top of usual Diabetes care compared to placebo on CIMT and definite coronary heart disease (CHD). Bezafibrate was not associated with improvements in CIMT versus placebo (change in CIMT 0.06 ± 0.38. versus 0.02 ± 0.41, *P* = .5). However, there was a significantly lower 3-year cumulative incidence rate of definite adverse CHD event in the bezafibrate treated group than in the placebo group (7 versus 23%, *P* = .01 log-rank test).

#### 2.2.3. Acyl-CoA: Cholesterol Acyltransferase (ACAT) Inhibitors

Two forms of ACAT have been identified. ACAT1 is found predominantly in macrophages, and ACAT2 is present in the liver and in the intestinal mucosa. Inhibition of ACAT1 is intended to make more free cholesterol available for reverse cholesterol transport, which, theoretically, could reduce lipid accumulation within atherosclerotic lesions and potentially influence progression of CAD. To evaluate the effect of ACAT inhibition on human coronary arteries, the ACAT Intravascular Atherosclerosis Treatment Evaluation (ACTIVATE) [48] enrolled 534 patients with symptomatic angiographically documented CAD and performed outset IVUS. Patients received usual care for secondary prevention, including statins. Patients were randomly assigned to receive the ACAT inhibitor pactimibe (100 mg per day) or matching placebo. The change in percent atheroma volume in 408 patients who completed the study at 18 months [27] was similar in the pactimibe and placebo groups (0.69 percent and 0.59 percent, resp.; *P* = .77). However, the total atheroma volume showed significant regression in the placebo group (−5.6 mm^3^, *P* = .001) but not in the pactimibe group (−1.3 mm^3^, *P* = .39); *P* = .03 for the comparison between groups. The combined incidence of adverse cardiovascular outcomes was similar in the two groups (*P* = .53).

A similar result was obtained with the ACAT inhibitor avasimibe. In the Avasimibe and Progression of coronary Lesions assessed by intravascular UltraSound (A-PLUS) clinical trial [49, 50], IVUS and coronary angiography were performed at baseline and repeated after up to 24 months of treatment. Approximately equal percentages of patients across groups (placebo, 50 mg, 250 mg, and 750 mg of avasimibe) received concurrent statin therapy (87% to 89%). Percent atheroma volume increased by 0.4% with placebo and by 0.7%, 0.8%, and 1.0% in the respective avasimibe groups (*P* = NS). LDL cholesterol increased during the study by 1.7% with placebo but by 7.8%, 9.1%, and 10.9% in the respective avasimibe groups (*P* < .05 in all groups). The negative effect of ACAT inhibitors on atherosclerosis progression was demonstrated in the terminated Familial hypercholesterolemia CIMT trial, the CAPTIVATE (Efficacy and Safety of the ACAT Inhibitor CS-505 [Pactimibe] for Reducing the Progression of Carotid Artery Disease) study, the group on statin alone had 3.4% CVD events compared with 6.3% (*P* = .02) in those on statin plus the ACAT inhibitor pactimibe. The primary and secondary CIMT end points were all consistent with worsening of atherosclerosis with pactimibe.

#### 2.2.4. Apo A-1 Milano

Apo A-1 is the major apolipoprotein component of HDL. Patients with Apo A-1 Milano mutation (cysteine for arginine at position 173), identified from rural Italy, characteristically have very low HDL cholesterol and high triglyceride levels. Paradoxically, these patients have no evidence of CAD. The infusion of Apo A-1 Milano-phospholipid complex significantly reduced intimal thickening and macrophage content in cholesterol fed rabbits [51]. This intervention was replicated in patients with ACS where the anti-atherosclerotic effect of intravenous recombinant ApoA-I Milano/phospholipid complexes (ETC-216) on atheroma burden was assessed [52]. ETC-216 weekly infusion for 5 weeks resulted in a decrement in mean percentage atheroma volume in the ETC-216 group and increased in the placebo group (−1.06% ± 3.17%; *P* = .02 from baseline and +0.14% ± 3.09%; *P* = .97 from baseline, resp.).

#### 2.2.5. Direct Lipoprotein-Associated Phospholipase A_2_ Inhibitor (Darapladib)

The presence of inflammatory cells and markers in the cap of atherosclerotic plaque correlates with increased risk of plaque rupture [53] and subsequent clinical events. Lipoprotein-associated phospholipase A2 is a hydrolytic enzyme that may play a role in membrane bound LDL modification [54]. A recent trial [55] showed lipoprotein-associated phospholipase A_2_ to be a novel risk factor independent of markers of inflammation or classic risk factors. An IVUS-based study conducted recently in 330 patients with angiographically documented CAD comparing the effects of the direct lipoprotein-associated phospholipase A_2_ inhibitor (Darapladib) to placebo showed that necrotic core volume measured by IVUS-radiofrequency (RF) increased significantly (4.5 ± 17.9 mm^3^; *P* = .009) in the control group, whereas active treatment with 160 mg of darapladib for 12 months halted this increase (−0.5 ± 13.9 mm^3^; *P* = .71), (difference of −5.2 mm^3^ between groups; *P* = .012). These intraplaque compositional changes occurred without a significant treatment difference in total atheroma volume measured by conventional IVUS (*P* = .95).

### 2.3. Antioxidants (Table 3)

Oxygen-free radicals can stimulate smooth muscle cells to proliferate and maybe induce in-stent restenosis after balloon angioplasty. Some antioxidant agents such as vitamins, probucol, and AGI-1067 were studied with varying anti-atherosclerotic effects.

#### 2.3.1. Antioxidant Vitamins

 Treatment with the anti-oxidant vitamins C and E to reduce coronary events and atherosclerosis progression has been controversial. The Vitamin E Atherosclerosis Progression Study (VEAPS) [56] evaluated the effect of vitamin E supplementation in 353 subjects on the change in CIMT. At 3-year follow-up, vitamin E supplementation failed to reduce the progression of CIMT (placebo: 0.0023 ± 0.007 mm/yr versus vitamin E: 0.0040 ± 0.0007 mm/yr; *P* = .08). The Antioxidant Supplementation in Atherosclerosis Prevention (ASAP) study [57] evaluated the effect of supplementation with vitamin E plus slow-release vitamin C on carotid atherosclerotic progression in 520 hypercholesterolemic middle-aged patients. At 6-year follow-up, supplementation significantly reduced the progression of intima-media thickness by 26% (*P* = .014). 

The antiatherosclerotic effects of antioxidant vitamins were studied in cardiac transplant patients as well [58, 59]. The study by Fang et al. [59] enrolled 40 cardiac transplant patients revealed that treatment with antioxidant vitamins C and E for 1 year retarded the progression of transplant-associated arteriosclerosis. The intimal index (plaque area/vessel area) measured by IVUS increased in the placebo group by 8% but did not change significantly in the treatment group (0.8%; *P* = .008). These promising results however, were questioned by Brown et al. [35]. In his evaluation of the effect of Simvastatin plus Niacin with or without antioxidant vitamins versus placebo, he reported that the regression in average stenosis induced by Simvastatin-niacin combination was reversed to a progression with the concomitant use of antioxidants (−0.4% with simvastatin-niacin alone versus +0.7% with simvastatin-niacin plus antioxidants; *P* = .02).

#### 2.3.2. Probucol and AGI-1067

 Probucol is an anti-hyperlipidemic drug that lowers the level of cholesterol in the bloodstream by increasing the rate of LDL catabolism. Additionally, probucol may inhibit cholesterol synthesis and delay cholesterol absorption. Probucol is a powerful antioxidant as well, which inhibits the oxidation of cholesterol in LDLs; this slows the formation of foam cells, which contribute to atherosclerotic plaques. Quantitative coronary angiography and IVUS based-studies that looked at probucol's effect on atheroma volume progression/regression revealed varying results [60, 61]. The first double-blind placebo controlled study failed to show a reduction in neointimal hyperplasia detected by IVUS after 6 months of treatment with probucol versus placebo (40.3 ± 26.7 mm^3^ versus 44.8 ± 39.3 mm^3^; *P* = .72) nor in restenosis rate detected by QCA (probucol: 19.4% versus placebo: 18.5%; *P* = .75) [60]. The second study conducted by Tardif et al. showed an increment in luminal area at PCI site versus placebo (3.69 ± 2.69 mm^2^ versus 2.66 ± 1.58 mm^2^; *P* ≤ .05) after 6 months of therapy with probucol on IVUS at the expense of a significant increase in QTc interval (increase in QTc >60 ms: placebo = 4.8% versus probucol = 17.4%; *P* = .02) [61]. AGI-1067 is a metabolically stable modification of probucol and an equipotent antioxidant to probucol. In a 1-year, IVUS-based, placebo-controlled trial, AGI-1067 was shown to cause a trend towards atheroma regression versus placebo in 232 patients (plaque volume: placebo: −0.4 mm^3^, *P* = .85 from baseline versus AGI-1067 −.04 mm^3^, *P* = .001 from baseline; *P* = .12 versus placebo) [62]. In the same study conducted by Tardif above, 280 mg of daily AGI-1067 increased luminal area at PCI site versus placebo in a dose response manner (3.6 ± 2.12 mm^3^ versus 2.66 ± 1.58 mm^3^; *P* ≤ .05) and did not increase the QTc interval [61].

### 2.4. Pleiotropic Effects of Other Medications (Table 4) 

#### 2.4.1. Cannabinoid Receptor Blockade

CB1 receptors, which are part of the endocannabinoid (EC) system, are integral in the metabolism of glucose and lipids. Blockade of this system causes decreased LDL cholesterol, elevated HDL cholesterol, decreased systolic blood pressure, decreased CRP, and decreased HbA1c [63–66]. The anti-atherosclerotic effect of CB1 blockade in abdominally obese patients with metabolic syndrome and pre-existing coronary disease was examined in the STRADIVARIUS study [67]. 839 patients were randomized to placebo or rimonabant 20 mg and underwent IVUS before and after 18 months of their randomized treatment, 676 patients completed the trial. There were significant reductions in body weight, waist circumference, triglycerides and C-reactive protein (CRP) in those treated with rimonabant. In addition, the rimonabant treated group had a significant increase in HDL-cholesterol. The study did not demonstrate an effect on percent atheroma volume (increase by 0.25% and 0.51%; *P* = .22) in the rimonabant and placebo groups, respectively. However, it did show a favorable effect on total atheroma volume (−2.2 mm^3^ in rimonbant group versus +0.88 mm^3^ in placebo group; *P* = .03). However, rimonabant did not demonstrate the risk/benefit profile that would enable it to be approved by the food and drug administration (FDA). The increased risk of neurological and psychiatric side effects—seizures, depression, anxiety, insomnia, aggressiveness, and more importantly suicidal thoughts among patients randomized to rimonabant warranted this decision. 

#### 2.4.2. The Insulin Sensitizers: Thiazolidinediones

 The oral hypoglycemic agent, pioglitazone, has been recently shown to possess anti-atherosclerotic activity. The Comparison of pioglitazone versus glimepiride on progression of coronary atherosclerosis in patients with type-2 diabetes; the PERISCOPE trial [68], randomized 543 patients with CAD and type-2 diabetes to receive one of the two commonly prescribed oral hypoglycemic agents, Pioglitazone or Glimipride. IVUS was done at study outset and then repeated after 18 months of treatment (*n* = 360) to compare the antiatherosclerotic effects of pioglitazone versus glimipride. The Change in percent atheroma volume from baseline increased by 0.73% (95% CI, 0.33% to 1.12%) with glimepiride and decreased by 0.16% (95% CI, −0.57% to 0.25%) with pioglitazone (*P* = .002). There was a significant improvement in HbA(1c) levels, HDL, and triglyceride in the pioglitazone versus glimipride group. 

The CHICAGO (Carotid Intima-media Thickness in Atherosclerosis Using Pioglitazone) study evaluated the role of pioglitazone on the progression of atherosclerosis in the carotids of 462 patients with type 2 diabetes. The rate of progression of CIMT was slowed by treatment with pioglitazone versus glimipride (−0.001 mm versus +0.012 mm respectively; *P* = .02) at all time points during the 72-week follow-up period [69].

## 3. Discussion and Conclusion

The primary interest of cardiovascular researchers in surrogate end points as a proxy for clinical outcomes stems from the fact that the evaluation of the effect of treatment on surrogate outcomes is often quicker and requires a smaller number of patients to demonstrate. The use of these surrogate endpoints however has been recently criticized because at the time of FDA approval, information remains incomplete about idiosyncratic reactions, off-target effects or delayed adverse effects. Therefore, the reward of rapid approval that turn out to be safe and effective needs to be balanced against harms that might occur later when drugs approved on the basis of surrogate end points turn out to have significant safety problems or to lack efficacy. Besides, the clearly recognized inherent limitations of noninvasive imaging modalities as well as quantitative coronary angiography in providing an accurate estimate of plaque burden can clearly distort the correlation of surrogate endpoints and clinical outcomes. Among the current imaging modalities, intravascular ultrasound remains the mainstay technique in assessing plaque progression/regression because it produces high quality images of the coronary lumen, vessel wall, and early atherosclerotic plaques with quantitative identification of all “atheroma components” and is capable of correlating increments in atheroma volume to future MACE. However, IVUS remains an invasive imaging modality with limited access in some catheterization labs and despite the good quality images it provides, it does not overcome the inherent limitation of surrogate endpoints and medication-related adverse events highlighted above. Moreover, the discrepancy between the results of the conventional IVUS and IVUS radiofrequency measurements inferred from the aforementioned “darapladib study,” warrants further research into which outcome measure to use and which one translates into adverse clinical outcomes. Therefore, given all the current limitations in the different imaging modalities available to quantify plaque volume and composition, and the intrinsic limitations with surrogate endpoints, one should be careful in applying the results of surrogate endpoint trials in patient care. Astute cardiovascular researchers are currently using the available imaging modalities in studying the pleiotropic effects of FDA-approved medications that possess good safety profile through the use of surrogate endpoints that will hopefully translate to beneficial clinical outcomes and add to the on-label usage of medications (such as pioglitazone use on top of conventional therapy in diabetic patients with CAD). Saying all that, the use of surrogate endpoints in assessing the efficacy of *novel *pharmacologic therapies in reducing adverse clinical cardiovascular outcomes remains controversial. 

## Figures and Tables

**Table 1:** Summary of trials highlighting the nonhemodynamic effects of different classes of antihypertensive medications.

Study	*N*	Medication	Imaging modality	Follow-up (months)	Outcome	Result		*P*-value
PREVENT [9]	825	Amlodipine versus placebo	QCA	36	Progression of coronary atherosclerosis (mm)	−0.095 versus −0.084		.38^‡^

PREVENT-substudy [9]	377	Amlodipine versus placebo	B-mode ultrasound	36	CIMT (mm)	−0.013 versus +0.033		.007^‡^

CAMELOT- NORMALIZE [13, 29]	274	Amlodipine versus placebo	IVUS	24	Δ PAV (%)	−0.8; *P* = .12	+0.3% (Favors Amlodipine)	.59^‡^
CAMELOT- NORMALIZE [13, 29]	274	Enalapril versus placebo	IVUS	24	Δ PAV (%)	−0.5; *P* = .32		

	617		B-mode ultrasound	24	Carotid wall thickness (mm)	0.82 versus 0.81		0.58^‡^
					Plaque score	11.1 versus 11.7		.93^‡^
Part-2 [15]		Ramipril versus placebo		48	Carotid wall thickness (mm)	0.83 versus 0.81		.58^‡^
					Plaque score	12 versus 13		.93^‡^

CAPARES [12]	635	Amlodipine versus Placebo	QCA	4	MLD (mm)	−0.30 ± 0.45 versus −0.29 ± 0.45		.84^‡^

SECURE [16]	732	Ramipril versus placebo	B-mode ultrasound	52	Δ CIMT (mm/yr)	0.0180 versus 0.0137 versus 0.0217		.033*

QUIET [17]	450	Quinapril versus placebo	QCA	36	Stenosis progression (%)	49 versus 47		NS^‡^
					Δ MLD index	−0.21 ± 0.03 versus −0.18 ± 0.03		NS
					Δ in % diameter stenosis index	+5.1 ± 1.0 versus 3.5 ± 1.0		NS

	394	Enalapril versus placebo	QCA	47.8	Δ mean diameter (mm)	−0.11 versus −0.11		NS
SCAT [18]					Δ minimum diameter (mm)	−0.12 versus −0.12		NS
					Δ % diameter stenosis (%)	+2.80 versus +2.90		NS

Waseda et. al. [21]	64	Olmesartan	IVUS	7	Vessel volume index (mm^2^)	9.9 ± 3.1 to 9.1 ± 2.7		<.01^¶^

MORE [20].	64	Olmesartan versus atenolol	B-ultrasound	24	CIMTin plaques >33.7 *μ*l (*μ*l)	−11.5 ± 4.4 versus +0.6 ± 2.5		.023

Nissen et. al. [14]	1515	Beta-blockers versus No BB	IVUS	18–24	Change in atheroma volume/yr (mm^3^/yr)	−2.4 ± 0.5 versus −0.4 ± 0.8		.034

*Ramipril 2.5 mg versus Ramipril 10 mg versus Placebo; *P*-value calculated across groups;

^*¶*^ Baseline versus Followup;

^†^ intimal index (plaque area/vessel area);

^‡^ difference between groups

**Table 2:** Summary of trials highlighting the pleiotropic effects of medications and treatment modalities used in the treatment of dyslipidemias.

Study	*N*	Medication	Imaging modality	Follow-up (months)	Outcome	Result	*P*-value
SCAT [18]	394	Simvastatin versus Placebo	QCA	47.8	Δ mean diameter (mm)	−0.07 versus −0.14	.004
					Δ minimum diameter (mm)	−0.09 versus −0.16	.001
					Δ % diameter stenosis (%)	+1.67 versus +3.83	.0003

Brown et. al. [35]	160	Simvastatin + niacin + antioxidants versus placebo	QCA	36	% diameter stenosis	+0.7 ± 3.2 versus +3.9 ± 5.2; *P* < .005	*P* = .02 for the difference b/w Simvastatin + Niacin + antioxidants and Simvastatin+Niacin alone
		Simvastatin + niacin versus placebo				−0.4 ± 2.8 versus +3.9 ± 5.2; *P* < .001	

CLAS [34]	162	Colestipol/niacin	QCA	24	% diameter stenosis (%)	0.3 ± 5.9 versus 2.7 ± 5.8	.02^¶^
					MLD (mm)	−0.01 ± 0.22 versus −0.09 ± 0.26	.04^¶^

REVERSAL [14]	654	80 mg atorvastatin versus 40 mg pravastatin	IVUS	18	Δ atheroma volume (%)	−0.4 versus +2.7	.02

METEOR [42]	984	Rosuvastatin versus placebo	B-ultrasound	24	Δ CIMT (mm/yr)	−0.0014 versus +0.0131	*P* < .001

ESTABLISH [36]	70	Atorvastatin versus placebo	IVUS	6	Δ plaque volume (%)	−13.1 ± 12.8 versus +8.7 ± 14.9	<.0001

ENHANCE [40]	720	Simvastatin versus Simvastatin + ezetimibe	B-ultrasound	24	Δ CIMT (mm)	0.0058 ± 0.0037 versus 0.0111 ± 0.0038	.29

SANDS [41]	499	Standard Rx : LDL <100 with statin alone	B-ultrasound	36	Δ CIMT (mm)	+0.039	*P* < .0001 between standard Rx. and aggressive Rx.
		Aggressive Rx : LDL <70 with Statin alone versus Statin + ezetimibe				−0.025 versus −0.012; *P* = .999	

LACMART [37]	18	LDL-apheresis + HMG-CoA	IVUS	12	Δ MLD (mm)	+0.12 versus −0.08	.008
		reductase I versus HMG-CoA reductase I			Δ plaque area (mm^2^)	−0.69 versus +0.88	.017

ASTEROID [28]	349	Rosuvastatin	IVUS	24	Mean Δ PAV (%)	−0.98 ± 3.15	<.001^¶^
					Median Δ total atheroma volume (%)	−6.8	<.001^¶^

Schartl et al. [39]	131	Atorvastatin versus	IVUS	12	Plaque volume (mm^3^)	1.2 ± 30.4 versus 9.6 ± 28.1	.191
		Usual care			Plaque echogenicity index (%)	42.2 versus 10.1	.021

DAIS [44]	731	Fenofibrate versus placebo	QCA	36	Δ MLD (mm)	−0.06 ± 0.01 versus −0.10 ± 0.016	.029

FIELD [45]	170	Fenofibrate versus placebo	CIMT	60	Δ CIMT (mm/yr)	0.140 versus 0.098	.722

Zhu et al. [46]	594	Fenofibrate versus placebo	CIMT	24	CIMT/D %	12.98 ± 2.62 versus 12.12 ± 2.26	*P* *<* .05

SENDCAP [47]	164	Bezafibrate versus placebo	CIMT	36	Δ CIMT (mm)	0.06 ± 0.38 versus 0.02 ± 0.41	*P* = .5

ACTIVATE [48]	534	ACAT inhibitor (pactimibe) versus placebo	IVUS	18	Δ PAV (%)	0.69 versus 0.59	.77
					Net Δ atheroma volume (mm^3^)	−1.3 versus −5.6	.03

A-PLUS [49, 50]	525	ACAT inhibitor (avasimibe) versus Placebo	IVUS	24	Δ PAV (%)	+0.4 versus +0.83	NS

Nissen et al. [52]	123	Recombinant Apo A-1 Milano/phospholipid complex (ETC-216) versus placebo	IVUS	5 wks	Δ PAV (%)	−1.06 ± 3.17	.02 (active)^ ¶^
						+0.14 ± 3.09	.97 (placebo)^ ¶^
					Δ atheroma volume (mm^3^)	−14.1	<.001^¶^

Surrey et al. [56]	330	Lipoprotein-associated phospholipase A_2_ inhibitor (Darapladib) versus placebo	IVUS	12	Δ Atheroma volume (mm^3^)	−4.9 ± 32.7 versus −5.0 ± 28.0	.95
			IVUS-RF		Δ Necrotic core volume (mm^3^)	−0.5 mm^3^; *P *= .71^¶^ versus +4.5; *P *= .009^¶^	.012

^¶^ Baseline versus Followup

**Table 3:** Summary of trials highlighting the anti-atherosclerotic effects of antioxidants.

Study	*N*	Medication	Imaging modality	Follow-up (months)	Outcome	Result	*P*-value
SECURE [16]	732	Vitamin E versus Placebo	B-mode ultrasound	52	CIMT (mm/yr)	0.0180 versus 0.0174	NS

ASAP [57]	520	Vitamin E + C	B-ultrasound	72	CIMT (%)	−26	.014^¶^

Fang et al. [59]	40	Vitamin E + C versus Placebo	IVUS	12	Δ average intimal index^†^ (%)	+0.8 versus +8	.008

VEAPS [56]	353	Vitamin E versus Placebo	B-ultrasound	36	Δ CIMT (mm/yr)	+0.0040 ± 0.0007 versus +0.0023 ± 0.0007	.08

Brown et al. [35]	160	Antioxidants versus Placebo	QCA	36	% diameter stenosis	+1.8 ± 4.2 versus +3.9 ± 5.2	NS
		Simvastatin + Niacin + antioxidants versus placebo				+0.7 ± 3.2 versus +3.9 ± 5.2	<.005

Nunes et al. [60]	54	Probucol versus placebo	IVUS	6	Intimal hyperplasia volume (mm^3^)	40.3 ± 26.7 versus 44.8 ± 28.3	.72
					% luminal volume obstruction	30.4 ± 14.5 versus 30.7 ± 17.2	.86
			QCA		Restenosis rate (%)	19.4 versus 18.5	.75

Tardif et al. [61]	305	Probucol versus placebo	IVUS	6	Luminal area @ PCI (mm^2^)	3.69 ± 2.69 versus 2.66 ± 1.58	<.05
		Succinobuccol (AGI-1067) versus placebo				3.36 ± 2.12 versus 2.66 ± 1.58	<.05

Tardif et al. [62]	232	280 mg Succinobuccol (AGI-1067) versus placebo	IVUS	12	Plaque volume (mm^3^)	−4.0; *P* =.001^¶^ versus −0.7; *P* = .85^*¶*^	.12^‡^

^¶^ Baseline versus Followup

^†^ intimal index (plaque area/vessel area)

^‡^ difference between groups

**Table 4:** Summary of trials highlighting the effects of oral hypoglycemic agents and CB1 receptor blockade on atherosclerosis. progression.

Study	*N*	Medication	Imaging modality	Follow-up (months)	Outcome	Result	*P*-value
PERISCOPE [68]	543	Pioglitazone versus Glimipride	IVUS	18	Δ PAV (%)	−0.16 versus +0.73	.002

CHICAGO [69]	462	Pioglitazone versus Glimipride	B-ultrasound	18	Δ CIMT (mm)	+0.012 versus −0.001	.02

STRADUVARIUS [67]	839	CB1-blockade (Rimonabant)	IVUS	18	Δ PAV (%)	+0.25 versus +0.51	.22
					Δ total atheroma volume (mm^3^)	−2.2 versus +0.88	.03

## References

[1] Wittes J, Lakatos E, Prostfield J (1989). Surrogate endpoints in clinical trials: cardiovascular diseases. *Statistics in Medicine*.

[2] Loscalzo J (2005). Clinical trials in cardiovascular medicine in an era of marginal benefit, bias, and hyperbole. *Circulation*.

[3] Böse D, von Birgelen C, Erbel R (2007). Intravascular ultrasound for the evaluation of therapies targeting coronary atherosclerosis. *Journal of the American College of Cardiology*.

[4] Waters D, Craven TE, Lesperance J (1993). Prognostic significance of progression of coronary atherosclerosis. *Circulation*.

[5] Willis AL, Nagel B, Churchill V (1985). Antiatherosclerotic effects of nicardipine and nifedipine in cholesterol-fed rabbits. *Arteriosclerosis*.

[6] Waters D, Lesperance J, Francetich M (1990). A controlled clinical trial to assess the effect of a calcium channel blocker on the progression of coronary atherosclerosis. *Circulation*.

[7] Borhani NO (1994). MIDAS: rationale, design and descriptive data of trial patients. The MIDAS Research Group. *Blood Pressure, Supplement*.

[8] Borhani NO, Brugger SB, Byington RP (1990). Multicenter study with isradipine and diuretics against atherosclerosis. US MIDAS Research Group. *Journal of Cardiovascular Pharmacology*.

[9] Mancini GBJ (2000). Overview of the prospective randomized evaluation of the vascular effects of Norvasc (amlodipine) trial: PREVENT. *Canadian Journal of Cardiology*.

[10] Zanchetti A, Agabiti Rosei E, Dal Palù C, Leonetti G, Magnani B, Pessina A (1998). The Verapamil in Hypertension and Atherosclerosis Study (VHAS): results of long-term randomized treatment with either verapamil or chlorthalidone on carotid intima-media thickness. *Journal of Hypertension*.

[11] Zannad F (2000). Effects of calcium antagonists on the progression of atherosclerosis and intima media thickness. *Drugs*.

[12] Thaulow E, Jørgensen B (2000). Results and clinical implications of the CAPARES trial. *Canadian Journal of Cardiology*.

[13] Brener SJ, Ivanc TB, Poliszczuk R (2006). Antihypertensive therapy and regression of coronary artery disease: insights from the comparison of Amlodipine versus Enalapril to Limit Occurrences of Thrombosis (CAMELOT) and Norvasc for Regression of Manifest Atherosclerotic Lesions by Intravascular Sonographic Evaluation (NORMALISE) trials. *American Heart Journal*.

[14] Nissen SE, Tuzcu EM, Schoenhagen P (2004). Effect of intensive compared with moderate lipid-lowering therapy on progression of coronary atherosclerosis. A randomized controlled trial. *Journal of the American Medical Association*.

[15] MacMahon S, Sharpe N, Gamble G (2000). Randomized, placebo-controlled trial of the angiotensin-converting enzyme inhibitor, ramipril, in patients with coronary or other occlusive arterial disease—part-2: Collaborative Research Group. Prevention of Atherosclerosis with Ramipril. *Journal of the American College of Cardiology*.

[16] Lonn EM, Yusuf S, Dzavik V (2001). Effects of ramipril and vitamin E on atherosclerosis: the study to evaluate carotid ultrasound changes in patients treated with ramipril and vitamin E (SECURE). *Circulation*.

[17] Cashin-Hemphill L, Holmvang G, Chan RC, Pitt B, Dinsmore RE, Lees RS (1999). Angiotensin-converting enzyme inhibition as antiatherosclerotic therapy: no answer yet. QUIET Investigators. *American Journal of Cardiology*.

[18] Teo KK, Burton JR, Buller CE (2000). Long-term effects of cholesterol lowering and angiotensin-converting enzyme inhibition on coronary atherosclerosis: the Simvastatin/Enalapril Coronary Atherosclerosis Trial (SCAT). *Circulation*.

[19] Takai S, Miyazaki M (2006). Effect of olmesartan medoxomil on atherosclerosis: clinical implications of the emerging evidence. *American Journal of Cardiovascular Drugs*.

[20] Stumpe KO, Agabiti-Rosei E, Zielinski T (2007). Carotid intima-media thickness and plaque volume changes following 2-year angiotensin II-receptor blockade. The Multicentre Olmesartan atherosclerosis Regression Evaluation (MORE) study. *Therapeutic Advances in Cardiovascular Disease*.

[21] Waseda K, Ozaki Y, Takashima H (2006). Impact of angiotensin II receptor blockers on the progression and regression of coronary atherosclerosis—an intravascular ultrasound study. *Circulation Journal*.

[22] (1982). A randomized trial of propranolol in patients with acute myocardial infarction. I. Mortality results. *Journal of the American Medical Association*.

[23] Olsson G, Rehnqvist N, Sjogren A (1985). Long-term treatment with metoprolol after myocardial infarction: effect on 3 year mortality and morbidity. *Journal of the American College of Cardiology*.

[24] Julian DG, Jackson FS, Szekely P, Prescott RJ (1983). A controlled trial of sotalol for 1 year after myocardial infarction. *Circulation*.

[25] (1981). Timolol-induced reduction in mortality and reinfarction in patients surviving acute myocardial infarction. *New England Journal of Medicine*.

[26] Sipahi I, Tuzcu EM, Wolski KE (2007). *β*-blockers and progression of coronary atherosclerosis: pooled analysis of 4 intravascular ultrasonography trials. *Annals of Internal Medicine*.

[27] Nissen SE, Tuzcu EM, Brewer HB (2006). Effect of ACAT inhibition on the progression of coronary atherosclerosis. *New England Journal of Medicine*.

[28] Nissen SE, Nicholls SJ, Sipahi I (2006). Effect of very high-intensity statin therapy on regression of coronary atherosclerosis: the ASTEROID trial. *Journal of the American Medical Association*.

[29] Nissen SE, Tuzcu EM, Libby P (2004). Effect of antihypertensive agents on cardiovascular events in patients with coronary disease and normal blood pressure. The CAMELOT study: a randomized controlled trial. *Journal of the American Medical Association*.

[30] Stier CT, Chander PN, Rocha R (2002). Aldosterone as a mediator in cardiovascular injury. *Cardiology in Review*.

[31] Duprez D, De Buyzere M, Rietzschel ER, Clement DL (2000). Aldosterone and vascular damage. *Current Hypertension Reports*.

[32] Takai S, Jin D, Muramatsu M, Kirimura K, Sakonjo H, Miyazaki M (2005). Eplerenone inhibits atherosclerosis in nonhuman primates. *Hypertension*.

[33] Von Birgelen C, Hartmann M, Mintz GS, Baumgart D, Schmermund A, Erbel R (2003). Relation between progression and regression of atherosclerotic left main coronary artery disease and serum cholesterol levels as assessed with serial long-term (≥12 months) follow-up intravascular ultrasound. *Circulation*.

[34] Azen SP, Mack WJ, Cashin-Hemphill L (1996). Progression of coronary artery disease predicts clinical coronary events: long-term follow-up from the cholesterol lowering atherosclerosis study. *Circulation*.

[35] Brown BG, Zhao X-Q, Chait A (2001). Simvastatin and niacin, antioxidant vitamins, or the combination for the prevention of coronary disease. *New England Journal of Medicine*.

[36] Okazaki S, Yokoyama T, Miyauchi K (2004). Early statin treatment in patients with acute coronary syndrome: demonstration of the beneficial effect on atherosclerotic lesions by serial volumetric intravascular ultrasound analysis during half a year after coronary event: the ESTABLISH study. *Circulation*.

[37] Matsuzaki M, Hiramori K, Imaizumi T (2002). Intravascular ultrasound evaluation of coronary plaque regression by low density lipoprotein-apheresis in familial hypercholesterolemia: the Low Density Lipoprotein-Apheresis Coronary Morphology and Reserve Trial (LACMART). *Journal of the American College of Cardiology*.

[38] Ballantyne CM, Raichlen JS, Nicholls SJ (2008). Effect of rosuvastatin therapy on coronary artery stenoses assessed by quantitative coronary angiography: a study to evaluate the effect of rosuvastatin on intravascular ultrasound-derived coronary atheroma burden. *Circulation*.

[39] Schartl M, Bocksch W, Koschyk DH (2001). Use of intravascular ultrasound to compare effects of different strategies of lipid-lowering therapy on plaque volume and composition in patients with coronary artery disease. *Circulation*.

[40] Kastelein JJP, Akdim F, Stroes ESG (2008). Simvastatin with or without ezetimibe in familial hypercholesterolemia. *New England Journal of Medicine*.

[41] Fleg JL, Mete M, Howard BV (2008). Effect of statins alone versus statins plus ezetimibe on carotid atherosclerosis in type 2 diabetes: the SANDS (Stop Atherosclerosis in Native Diabetics Study) trial. *Journal of the American College of Cardiology*.

[42] Crouse JR, Grobbee DE, O’Leary DH (2004). Measuring effects on intima media thickness: an evaluation of rosuvastatin in subclinical atherosclerosis—the rationale and methodology of the METEOR study. *Cardiovascular Drugs and Therapy*.

[43] Taylor AJ, Villines TC, Stanek EJ (2009). Extended-release niacin or ezetimibe and carotid intima-media thickness. *New England Journal of Medicine*.

[44] (2001). Effect of fenofibrate on progression of coronary-artery disease in type 2 diabetes: the Diabetes Atherosclerosis Intervention Study, a randomised study. *Lancet*.

[45] Hiukka A, Westerbacka J, Leinonen ES (2008). Long-term effects of fenofibrate on carotid intima-media thickness and augmentation index in subjects with type 2 diabetes mellitus. *Journal of the American College of Cardiology*.

[46] Zhu S, Su G, Meng QH (2006). Inhibitory effects of micronized fenofibrate on carotid atherosclerosis in patients with essential hypertension. *Clinical Chemistry*.

[47] Elkeles RS, Diamond JR, Poulter C (1998). Cardiovascular outcomes in type 2 diabetes: a double-blind placebo- controlled study of bezafibrate: the St. Mary’s, Ealing, Northwick Park Diabetes Cardiovascular Disease Prevention (SENDCAP) Study. *Diabetes Care*.

[48] Nicholls SJ, Sipahi I, Schoenhagen P (2006). Intravascular ultrasound assessment of novel antiatherosclerotic therapies: rationale and design of the Acyl-CoA:Cholesterol Acyltransferase Intravascular Atherosclerosis Treatment Evaluation (ACTIVATE) Study. *American Heart Journal*.

[49] Tardif J-C, Grégoire J, Lespérance J (2002). Design features of the Avasimibe and Progression of coronary Lesions assessed by intravascular UltraSound (A-PLUS) clinical trial. *American Heart Journal*.

[50] Tardif J-C, Grégoire J, L’Allier PL (2004). Effects of the acyl coenzyme A:cholesterol acyltransferase inhibitor avasimibe on human atherosclerotic lesions. *Circulation*.

[51] Ameli S, Hultgardh-Nilsson A, Cercek B (1994). Recombinant apolipoprotein A-I Milano reduces intimal thickening after balloon injury in hypercholesterolemic rabbits. *Circulation*.

[52] Nissen SE, Tsunoda T, Tuzcu EM (2003). Effect of recombinant ApoA-I Milano on coronary atherosclerosis in patients with acute coronary syndromes: a randomized controlled trial. *Journal of the American Medical Association*.

[53] Davies MJ, Richardson PD, Woolf N, Katz DR, Mann J (1993). Risk of thrombosis in human atherosclerotic plaques: role of extracellular lipid, macrophage, and smooth muscle cell content. *British Heart Journal*.

[54] Leitinger N, Watson AD, Hama SY (1999). Role of group II secretory phospholipase A2 in atherosclerosis: 2. Potential involvement of biologically active oxidized phospholipids. *Arteriosclerosis, Thrombosis, and Vascular Biology*.

[55] Packard CJ, O'Reilly DSJ, Caslake MJ (2000). Lipoprotein-associated phospholipase A2 as an independent predictor of coronary heart disease. West of Scotland Coronary Prevention Study Group. *New England Journal of Medicine*.

[56] Hodis HN, Mack WJ, LaBree L (2002). Alpha-tocopherol supplementation in healthy individuals reduces low-density lipoprotein oxidation but not atherosclerosis: the Vitamin E Atherosclerosis Prevention Study (VEAPS). *Circulation*.

[57] Salonen RM, Nyyssönen K, Kaikkonen J (2003). Six-year effect of combined vitamin C and E supplementation on atherosclerotic progression: the Antioxidant Supplementation in Atherosclerosis Prevention (ASAP) Study. *Circulation*.

[58] Behrendt D, Beltrame J, Hikiti H (2006). Impact of coronary endothelial function on the progression of cardiac transplant-associated arteriosclerosis: effect of anti-oxidant vitamins C and E. *Journal of Heart and Lung Transplantation*.

[59] Fang JC, Kinlay S, Beltrame J (2002). Effect of vitamins C and E on progression of transplant-associated arteriosclerosis: a randomised trial. *Lancet*.

[60] Nunes GL, Abizaid AC, Theodoro MP (2006). Role of probucol in inhibiting intimal hyperplasia after coronary stent implantation: a randomized study. *American Heart Journal*.

[61] Tardif J-C, Grégoire J, Schwartz L (2003). Effects of AGI-1067 and probucol after percutaneous coronary interventions. *Circulation*.

[62] Tardif J-C, Grégoire J, L’Allier PL (2008). Effects of the antioxidant succinobucol (AGI-1067) on human atherosclerosis in a randomized clinical trial. *Atherosclerosis*.

[63] Després J-P, Golay A, Sjöström L (2005). Effects of rimonabant on metabolic risk factors in overweight patients with dyslipidemia. *New England Journal of Medicine*.

[64] Pi-Sunyer FX, Aronne LJ, Heshmati HM, Devin J, Rosenstock J (2006). Effect of rimonabant, a cannabinoid-1 receptor blocker, on weight and cardiometabolic risk factors in overweight or obese patients—RIO-North America: a randomized controlled trial. *Journal of the American Medical Association*.

[65] Scheen AJ (2007). Cannabinoid-1 receptor antagonists in type-2 diabetes. *Best Practice and Research: Clinical Endocrinology and Metabolism*.

[66] Van Gaal LF, Rissanen AM, Scheen AJ, Ziegler O, Rössner S (2005). Effects of the cannabinoid-1 receptor blocker rimonabant on weight reduction and cardiovascular risk factors in overweight patients: 1-year experience from the RIO-Europe study. *Lancet*.

[67] Nissen SE, Nicholls SJ, Wolski K (2008). Effect of rimonabant on progression of atherosclerosis in patients with abdominal obesity and coronary artery disease: the STRADIVARIUS randomized controlled trial. *Journal of the American Medical Association*.

[68] Nissen SE, Nicholls SJ, Wolski K (2008). Comparison of pioglitazone vs glimepiride on progression of coronary atherosclerosis in patients with type 2 diabetes: the PERISCOPE randomized controlled trial. *Journal of the American Medical Association*.

[69] Mazzone T, Meyer PM, Feinstein SB (2006). Effect of pioglitazone compared with glimepiride on carotid intima-media thickness in type 2 diabetes: a randomized trial. *Journal of the American Medical Association*.

